# One SQ HEDGES DNA vector only dose produces durable hGLA or anti-SARS-CoV-2 mAb therapeutic serum protein levels

**DOI:** 10.1371/journal.pone.0315041

**Published:** 2025-10-31

**Authors:** Alice Ye, Marissa Mack, Ryan Ice, Tim Heath, Elof Ericksson, Stanford Shoor, Robert D. Steiner, Chakkrapong Handumrongkul, Robert Debs

**Affiliations:** 1 DNARx, San Francisco, California, United States of America; 2 Dartmouth Medical Center 4, Hanover, New Hampshire, United States of America,; 3 Stanford University, Stanford, California, United States of America; 4 University of Wisconsin, Madison, Wisconsin, United States of America; Cairo University Faculty of Veterinary Medicine, EGYPT

## Abstract

It is estimated that ~2 billion people worldwide cannot afford even basic medicines. **B**ioreactor-produced **r**ecombinant **p**rotein **t**herapies (BRPTs), among the world’s most-expensive medicines, have revolutionized treatment of a wide-spectrum of human-diseases, particularly the ~ 7,000 incurable, rare human single-protein, monogenic-deficiency diseases. Currently, BRPT are limited by toxicity, immunogenicity, short protein T^1^/_2_s and high-cost, creating a worldwide access-gap between those who can afford BRPTs versus those who cannot. Fabry disease (FD) is a monogenic deficiency disease caused by pathogenic-variants of the galactosidase-a (GLA) gene. FD damages heart, kidneys, CNS, gastrointestinal tract, and eyes. State-of-the-art anti-FD therapy, hGLA enzyme-replacement-therapy (ERT), requires bi-weekly IV infusions lifelong, costing ~ $200,000 per-patient per-year. High-lifetime costs can cause significant numbers of FD patients to permanently discontinue hGLA-ERT, thereby accelerating FD progression, which can lead to premature-death. Subcutaneously administered plasmid DNA alone has not been previously reported to transfect subcutaneous fat. Here we show one **S**ubcutaneous **A**dministration of **H**EDGES **D**NA vectors **A**lone (**SAHDA**) encoding the wildtype hGLA protein safely produces durable hGLA serum protein levels in the 1–10 ng/mL normal human hGLA range in immunocompetent mice. Unexpectedly, one administration of a highly-optimized SAHDA version encoding hGLA produced durable, ~ 100-fold higher hGLA serum protein levels than the 10 ng/mL high-normal human level. Importantly, components of the SADHA platform can be simply-modified to control the level and duration of hGLA serum protein-levels produced over a broad temporal-range in mice. Furthermore, one SAHDA-based administration of a HEDGES DNA-vector encoding a highly-neutralizing anti-SARS-CoV-2 monoclonal antibody (mAb) safely produces long-term protective serum mAb levels in immunocompetent-mice. SAHDA offers multiple advantages over BRPT, including not requiring an intact cold-chain and being readily freeze-dried. This combination enables SAHDA’s rapid deployment, then prolonged storage at ambient temperatures, even in equatorial-areas worldwide. SAHDA can readily be self-administered worldwide. It also obviates severe intravenous-infusion reactions. Taken-together, SAHDA may more effectively-, safely-, durably-, and cost-effectively-treat a spectrum of now poorly-treatable diseases worldwide.

## Introduction

The great majority of human single protein deficiency disease patients, they comprise ~0.5% of the population, remain incurable [1,2]. When available, ERT is current state-of-the art therapy for these diseases [3,4]. However, ERT is almost never curative, requires frequent redosing, usually daily to once every two weeks for life, is inordinately expensive (from $40,000->$200,000 per-year per patient) as well as can cause life-threatening allergic infusion reactions [1,2,5,6]. AAV gene therapy has revolutionized the treatment of many up to now untreatable rare human monogenic deficiency diseases. However, AAV elicits potentially lethal human adaptive immune responses, cannot be effectively re-dosed, and can cause insertional mutagenesis, rarely leading to oncogenesis [7,8].

Fabry disease (FD), a lysosomal storage disorder, is a rare monogenic single protein deficiency disease caused by pathogenic variants in the *GLA* gene [9–13]. These variants lead to reduced GLA enzymatic activity, preventing the breakdown of glycolipids including globotriaosylceramide (Gb3) and deacylated globotriaosylsphingosine (lyso-GL-3). Clinically, FD damages the heart, kidneys, nervous system, gastrointestinal tract, and eyes among other tissues. Wild type hGLA protein has a short protein T^1^/_2_ of <20 minutes [14]. The normal human range of wildtype hGLA protein serum levels is from 1 to 10 ng per mL [9–13].

The development of bioreactor-produced recombinant protein therapies (BRPTs) has revolutionized the treatment of a progressively widening spectrum of previously difficult or impossible to treat human diseases [15,16]. However, to date, the promise of BRPT far exceeds its impact on human health worldwide [17]. Specifically, BRPTs are significantly limited by each of the following: All BRPTs must now be produced *ex viv*o in costly bioreactors, requiring the use of multiple different BRPT production systems. These include bacteria, insect cells, mammalian cells, transgenic animals, or plants. Specific production systems are chosen based on the requirements for specific protein modifications, (e.g., glycosylation, phosphorylation and/or proteolytic cleavage) to maximize biologic activity [14–16]. Additional challenges include protein solubility limitations as well as immunogenicity and toxicity issues [15,16,18]. Multiple different factors are responsible for the high costs incurred by BRPTs, a number of which cost ≥ $100,000 per patient per year for life [6,10–13]. These costs are derived from ongoing *ex vivo* BRPT production followed by costly post-production logistical challenges, including the continued requirement for an intact cold chain. Cold chain-imposed limitations complicate the worldwide distribution as well as storage of BRPT at all stages prior to their administration [15,16]. Not infrequently, BRPTs require intravenous administration, further increasing logistical issues, costs, and creating potentially significant toxicity risks [15,16]. Furthermore, BRPT T^1^/_2_s most often range from <20 minutes to ~four weeks [10–14]. Thus, individuals with rare human monogenic, single protein deficiency diseases, including FD, require frequent BRPT redosing for life [6,10–14]. The high, ongoing costs these patients must pay to afford life-enhancing or lifesaving BRPTs have created a persistent access gap between those who can afford BRPTs versus those who cannot. Of significant concern, FD can progress more rapidly in individuals unable to afford the high, ongoing costs imposed by hGLA enzyme replacement therapy (ERT). In some patients, these unaffordable costs result in their accelerated death [10–13,19]. In addition, IV hGLA ERT infusions can elicit recurrent, at times severe allergic reactions in FD patients, sometimes requiring them to discontinue hGLA ERT. Like hGLA ERT-related cost issues, discontinuing ERT due to recurrent allergic infusion reactions can also lead to more rapid disease progression, precipitating early death.

DNARx previously developed HEDGES, (**H**igh-level **E**xtended **D**uration **G**ene **E**xpression **S**ystem). HEDGES is a direct-in-body, sequential intravenous, nonviral, cationic plus neutral liposome then DNA-vector based gene therapy-based platform. Our first-generation HEDGES platform safely, rapidly, and durably produced a wide range of cDNA encoded proteins. These include FDA approved mAbs, as well as other FDA approved protein therapies [20]. HEDGES durably-produces biologically and therapeutically significant levels of DNA vector-encoded proteins following one administration into immunocompetent mice. Although HEDGES durably produces its cDNA encoded proteins, HEDGES duration of protein production in mice can be controlled to a significant degree by simply modifications of the liposomal lipid composition, the liposomal mean diameter, the liposome to DNA ratio, the DNA vector dose or the specific DNA promoter enhancer element incorporated into the DNA vector [20].

Specifically, one safe, IV administration of our first-generation HEDGES DNA vector encoding the wildtype human G-CSF (hG-CSF) protein (T^1^/_2_ ~ 2 hours) into immunocompetent CD-1 mice produced significantly elevated absolute neutrophil counts together with partial replacement of normal hematopoietic precursor cells by immature neutrophils in bone marrow and spleen [20]. This is the desired therapeutic response to recombinant hG-CSF protein therapy. Wildtype hG-CSF protein replacement therapy is now administered daily to treat a spectrum of human chronic neutropenic diseases [21]. Specifically, one intravenous (IV) HEDGES-based hG-CSF administration increased the duration of therapeutic hG-CSF serum protein levels produced by greater than 13,900-fold when compared to one administration of bioreactor-produced wildtype recombinant hG-CSF protein [20,21].

In addition, one IV administration of a HEDGES DNA vector encoding 5J8 [22], an anti-1918 pandemic-influenza mAb, rituximab [23], an anti-human CD20 mAb, or mepolizumab [24], an anti-human IL-5 mAb, each produced prolonged bioactive mAb serum levels. Furthermore, we showed that HEDGES DNA vectors neither detectably integrate into host genomic DNA nor elicit adaptive immune responses, thus enabling HEDGES to be effectively and durably re-expressed following re-dosing in immunocompetent mice [13]. Lastly, critical mouse toxicity markers remain at or near background levels following IV HEDGES administration [20].

Until now, direct percutaneous, subcutaneously administered naked DNA plasmid vectors alone directly into subcutaneous fat has not produced detectable gene expression [25]. Conversely, when electroporation is added to subcutaneous administration of plasmid DNA alone, intense gene expression is produced [25]. Here, we sought to create direct percutaneous **S**ubcutaneous **A**dministration of **H**EDGES **D**NA vectors **A**lone (SAHDA) as an effective, safe, durably expressing, highly cost-effective platform. If successful, SAHDA could significantly increase therapeutic efficacy, while concurrently reducing the toxicity, cost, and logistical constraints that now restrict BRPT’s impact worldwide. For example, one SAHDA administration of a HEDGES DNA vector encoding one of the ~ 7000 different now absent monogenic deficiency disease proteins may possibly persistently functionally cure at least one of these diseases [26]. Furthermore, SAHDA produced recombinant protein therapies (RPT) could offer significant safety as well as cost advantages over IV BRPT.

Given the substantial opportunities afforded by successfully creating the SAHDA platform, we anticipated it would be extremely difficult to create, for each of the following reasons: First, a previous attempt using subcutaneous injection of plasmid DNA alone encoding GFP into the interscapular fat pads of guinea pigs produced no detectable GFP expression when a GFP-encoding plasmid DNA vector alone was injected [25]. However, when electroporation was added to the identical DNA dose, subcutaneous fat produced intense GFP gene expression [25]. In addition, there is no clearly documented cellular receptor for naked DNA alone. To date, DNA alone appears unable to efficiently enter subcutaneous fat cells in the absence of an attached carrier molecule, electroporation, or hydrodynamic therapy [25,27–29]. We specifically designed SAHDA to lack any cationic carrier or ligand that could facilitate intracellular delivery of the HEDGES DNA vector. Furthermore, intramuscular (IM) injection of naked DNA vectors into muscle produces detectable, but neither biologically nor therapeutically relevant serum levels of cDNA-encoded genes. Like previous subcutaneous administration of naked plasmid DNA vectors, concurrent electroporation is required to produce biologically and/or therapeutically relevant serum levels of IM administered, plasmid DNA vector alone encoded genes [30]. Based on the above limitations, we prioritized the following experimental variables to test to maximize the possibility of creating an efficient, safe, cost-effective SAHDA platform. These variables included the design of the HEDGES DNA vectors injected, the DNA vector dose administered, the total diluent volume administered, the DNA vector dose to diluent volume ratio administered, the presence or absence of hyaluronidase as well as the total hyaluronidase dose administered, whether injected animals should be normal weight or obese, and the optimal site or sites of subcutaneous fat injected.

## Materials and methods

### Cell culture

Primary human white preadiopcytes (PromoCell, C-12735) were grown in preadipocyte growth medium (PromoCell, C-27410) at 37°C 5% CO_2_. Differentiated adipocytes were grown in adipocyte nutrition medium (C-27438) at 37°C 5% CO_2_. Cell culture medium was changed every 48–72 hours.

### Differentiation

Primary human preadipocytes (PromoCell, C-12735) were plated for differentiation at a concentration of 0.75 x10^5^ cells per well in a 24-well plate. Once the cells reached confluence, the growth medium was changed to preadipocyte differentiation medium (C-27436) and the cells were incubated 37°C 5% CO_2_ for 72 hours, then the medium was changed to adipocyte nutrition medium (C-27438) and replaced every 72 hours for 21 days.

### Transfection

Primary human white preadiopcytes (PromoCell, C-12735) were plated for transfection at a concentration of 0.25 x10^5^ cells per well in a 24-well plate. The cells were transfected with 1 µg plasmid DNA encoding an anti-CoV-2 mAb complexed with 0.54 µl/Expifectamine 293 (Thermo Fisher, A14524)/µg DNA. Thin-film transfections were performed by diluting the DNA and lipids in preadipocyte growth medium before combining and incubating 20 minutes at room temperature. Then 200 µl of DNA-lipid complexes were added to the plated cells that were washed with 1X PBS. The 24-well plate was incubated at 37°C 5% CO_2_ for 30 minutes. Following incubation, 1 mL preadipocyte growth medium was added to each well and the cells were incubated overnight at 37°C 5% CO_2_. The media was collected on day 4 post-transfection and assayed for hIgG levels. Each transfection is n = 2. For the transfection of fully differentiated adipocytes, Primary human white preadiopcytes (PromoCell, C-12735) were plated and differentiated as described above. Transfection was carried out via thin-film as described above. The media was collected on day 4 post-transfection and assayed for hIgG levels. Each transfection is n = 2. For the transfection of differentiating preadipocytes, the thin-film method was used as described above using 1 µg DNA and 0.54 µl Expifectamine, and transfection took place once every week on days 0, 6, 15, and 21. The supernatant was collected 72 hours post transfection and every 72 hours thereafter assayed for hIgG levels. Each transfection is n = 2.

### ELISA

For the hIgG specific ELISA, immunoassay plates were coated with goat anti-human IgG Fc capture antibody (Bethyl laboratories: A80-104A) at 2 µg/mL overnight and blocked with 2% bovine serum albumin. The samples were detected with goat anti-human IgG Fc, HRP (Millipore: AP113P) at 1:20000. The standard protein is native human IgG (BioRad: 5172-9017). The plates were developed using TMB ultra substrate (Fisher: PI34029). The absorbance at 450nm was measured on a BMG Labtech spectrostar nano plate reader and the standard curve was analyzed by 4PL using the MARS data analysis software. For the GLA ELISA, a commercial kit was used from RayBiotech (ELH-aGLA).

### Subcutaneous injections

For inguinal mammary fat pad injections: Rodents were anesthetized with isoflurane and shaved in the inguinal region. A needle was inserted from the caudal end of the abdomen under the skin up into the inguinal fat pad and the injection bolus deposited roughly half an inch from the puncture site. Rats were also injected in the 3^rd^ mammary fat pad using the same technique. Total injection volumes were divided evenly between the two sides.

For subscapular fat pad injections: Rodents were anesthetized with isoflurane. The skin under and behind the shoulder blades was lifted gently with tweezers and the needle inserted under the skin into the fat pads. The animal protocols were approved by the Institutional Animal Care and Use Committee at the California Pacific Medical Center Research Institute.

### Plasmid DNA

The CpG free and codon-optimized ORF of wild-type hGLA 1x hyFc and anti-CoV-2 mAb (CV07-209) heavy and light chain separated by P2A were synthesized by GeneArt (ThermoFisher, NY). The cDNAs were, then, inserted into our HEDGES expression vector as previously described [20]. The plasmids were grown in *E. coli* GT115 and isolated to verify by restriction enzyme and gel electrophoresis. The correct expression of plasmids was confirmed in small in vivo mice experiment via IV HEDGES tail vain injection. The large production and isolation of the plasmids were performed according to manufacture instruction using Qiagen endotoxin-free giga prep kit (Qiagen, MD). The plasmids were dissolved in 0.2 µM filtered and sterile lactated ringers. The endotoxin level was measured using chromogenic LAL assay (Genscript, NJ). The average endotoxin level is 0.01 EU/µg.

### Diluents, cationic lipids, and hyaluronidase

Isotonic Lactated Ringer’s was used for most injections. For hypotonic injections, isotonic LR and millipure water were mixed 1:2. For hypertonic injections, isotonic LR was mixed 1:2 with a 7.2% hypertonic saline. Cationic lipid used was DOTAP from Avanti Polar Lipids (890890) suspended in isotonic LR. Hyaluronidase (MP Biomedicals) was suspended in isotonic LR.

### Statistical analysis

The comparison of each two treatments was done in pair using Welch’s t-test where * p ≤ 0.05, ** p ≤ 0.01.

## Results

**Fig 1:**
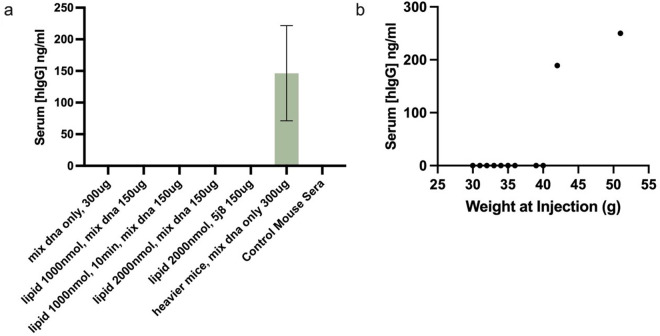
Direct percutaneous injection of naked DNA produces DNA vector-encoded protein. Groups of 3 CD-1 female mice were injected with either a HEDGES DNA vector alone at 1ug/uL in Lactated Ringer’s, or co-injected with both the DNA vector and DOTAP cationic lipid. a) serum levels of hIgG were measured at day 8 following SAHDA administration, graphed as mean ±SEM. b) weight at injection was correlated to serum hIgG serum levels at day 8.

To identify the subcutaneous fat pad injection site and methods that maximize the efficiency of SAHDA-based subcutaneous administration of HEDGES DNA vectors in mice, we tested direct percutaneous injection into both subcutaneous inguinal mammary fat pads (mammary fat pads 4 and 5), and/or into the subcutaneous subscapular fat pads. Mice were injected with HEDGES DNA vectors encoding a mAb, either mepolizumab [24] (anti-IL5) or 5J8 [22] (an anti-1918-pandemic influenza A mAb), suspended in Lactated Ringer’s (LR). The serum levels of hIgG were subsequently measured over time post administration. Additionally, mice were injected with either DNA alone or co-injected with various amounts of cationic lipid based on our previous IV HEDGES protocol utilizing both DNA vectors and cationic plus neutral lipids [20]. None of the subscapular injected mice produced detectable hIgG serum levels. However, one group of mice injected in the inguinal mammary fat pads with DNA alone produced serum hIgG levels significantly above background levels (Fig 1a). Provocatively, the single group of mice that produced significant serum hIgG levels weighed significantly more than any of the other mice included in this experiment (Fig 1b).

### SAHDA mediated subcutaneous production of significant hGLA serum levels

We next attempted to maximize the level and duration of SAHDA mediated serum production of a different cDNA encoded protein, human α-galactosidase enzyme (hGLA), using the same SAHDA administration technique. Pathogenic mutations in this gene are responsible for the lysosomal storage disorder Fabry disease (FD). The T^1^/_2_ of the endogenous hGLA protein is < 20 minutes, rendering durable single dose control of FD using current hGLA ERT impossible [10–12]. Thus, the long-term production of the wildtype GLA enzyme protein directly in the body should significantly improve the treatment of FD patients.

**Fig 2:**
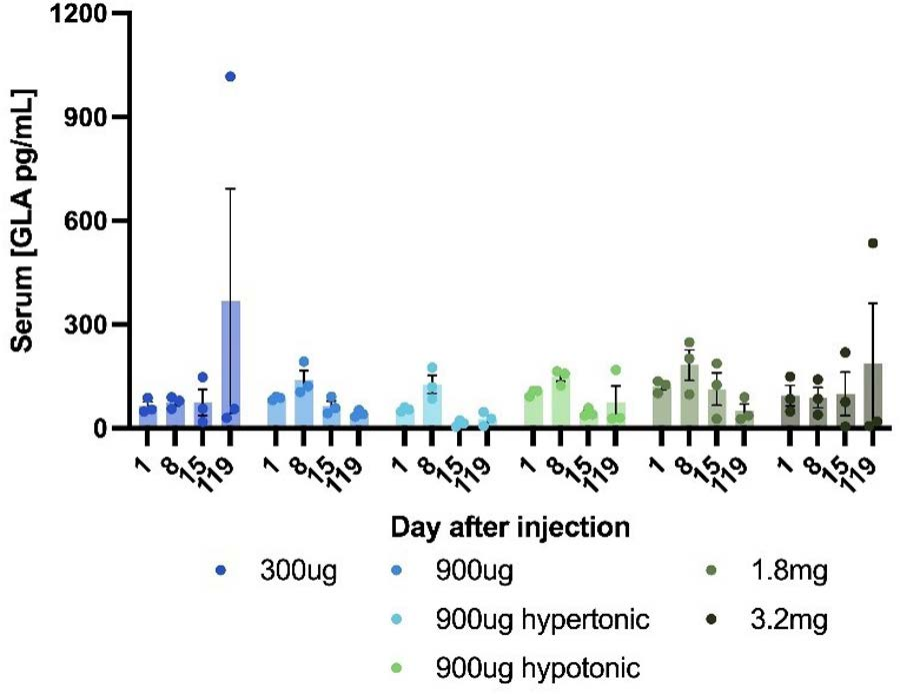
Subcutaneous DNA injection produces. Mice were injected subcutaneously with HEDGES DNA vector encoding hGLA in LR at 1ug/uL (300ug), 3ug/uL (900ug and 1.8 mg), or 6ug/uL (3.2 mg). Sera were collected and assessed by ELISA. Data are shown as mean ±SEM (n = 3).

Initially, we tested four different doses of a HEDGES DNA vector encoding wildtype hGLA. Only low level hGLA serum protein production was observed in all conditions tested from day 1 to day 15 post injection (Fig 2). Subsequently, the mice were reassessed a hundred days later at day 115 post-injection. This delay was necessitated by an extended transition period required to move these mice from the vivarium they initially occupied to a new vivarium at a different location. By day 115 post injection, two of the mouse groups were producing hGLA serum levels approaching therapeutic. Normal human serum hGLA levels are in the 1–10 ng/mL range [31]. This unexpected late rise in hGLA serum levels in two of these mouse groups revealed that this early version of the SAHDA platform required serial bleeds for significantly longer than 15 days post administration to be able to detect the production of SAHDA encoded proteins. It also demonstrated that, unlike IV HEDGES DNA vector administration, which almost inevitably produces bioactive as well as therapeutic serum levels of HEDGES encoded proteins within 24 hours post HEDGES injection [20]. SAHDA requires more time before producing biologically significant levels of SAHDA DNA vector-encoded proteins.

### Modifications to the SAHDA protocol substantially increased the level as well as duration of hGLA serum levels produced in mice

**Fig 3:**
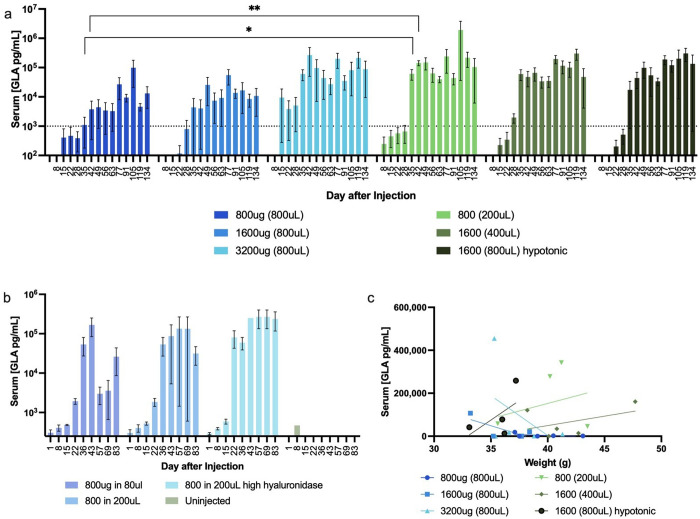
Single dose SAHDA DNA vector produces supratherapeutic hGLA serum levels. **(a)** Groups of 5 mice were injected with 0.8, 1.6, or 3.2 mg HEDGES DNA vector in volumes of isotonic or hypotonic LR shown, containing 0.3kU/kg hyaluronidase. Serum levels of hGLA protein measured over time by ELISA. Significance assessed by Welch’s t-test * p < 0.05, ** p < 0.01. **b)** Groups of 3 mice were injected with the same vector in isotonic LR with the same dose of hyaluronidase as before or 2kU/kg. **c)** Serum levels of hGLA at day 49 after injection of the individual mice in part a) correlated with weight in grams. All data shown are mean ±SEM.

We subsequently administered SAHDA exclusively into the inguinal mammary fat pads. We then injected groups of mice with three different DNA doses in varying volumes of LR, each containing 0.3kU/kg hyaluronidase. Hyaluronidase is an enzyme commonly used to increase absorption of treatments given subcutaneously [32]. Additionally, a group of mice was injected with a HEDGES hGLA DNA vector suspended in hypotonic LR instead of isotonic LR. In contrast to the prior experiment, where serum hGLA levels remained at background levels until at least until day 15 post injection, in this experiment hGLA serum levels significantly above background appeared in one group by day 8 post injection. Other groups produced highly significant hGLA serum levels in the subsequent few weeks (Fig 3a). Injecting the 800uL LR volume, the high DNA dose of 3200ug produced significant serum hGLA levels as early as day 15 post injection. hGLA serum levels subsequently remained within the 1–10 ng/mL normal human hGLA range for greater than the next 133 mouse days. Using the 800uL diluent volume, the two lower DNA vector doses (800ug and 1600ug) produced hGLA serum levels in the normal human range by day 35 post administration. Interestingly, both these two lower HEDGES DNA vector doses also produced serum hGLA levels in the normal human range when used in lower LR diluent volumes. In addition, the 800ug DNA dose diluted in 200uL LR produced significantly higher serum hGLA levels at the first two bleed timepoints, reaching therapeutic serum hGLA levels at the earlier time points at days 35 and 42 respectively when compared to the same DNA dose administered in higher diluent volumes. Strikingly, by day 42 post one SAHDA administration, four of the SAHDA-injected groups produced at least two orders of magnitude higher hGLA serum levels (~1000 ng/mL) than the upper limit of normal human serum hGLA levels (10 ng/mL). As documented previously, the duration of HEDGES protein production can be controlled to a significant extent simply by modifying the liposomal lipid composition, the liposomal mean diameter, the DNA vector dose or the specific DNA promoter enhancer element incorporated into the DNA vector [20].

We then assessed whether further reducing the LR volume or increasing the dose of hyaluronidase to 2kU/kg while retaining the same 800ug HEDGES DNA vector dose would produce earlier and/or higher peak hGLA serum levels. While further decreasing LR diluent volume from 200uL to 80uL did not further increase hGLA serum levels, increasing the hyaluronidase dose did produce an earlier rise of hGLA serum levels to peak levels by day 22 rather than at day 35 (Fig 3b). Thereafter, mouse sera remained consistently at or approaching 1000 ng/mL hGLA serum levels (~100 fold higher than the upper normal range for human hGLA serum levels) for greater than the next 100 mouse days. Thus, one SAHDA administration encoding hGLA produced hGLA serum protein levels at or approaching 1000 ng/mL for greater than the next hundred mouse days. Therefore, one SAHDA-based administration of a HEDGES DNA vector encoding hGLA produced durable hGLA serum protein levels >100 ng/mL, a greater than 21,600-fold duration increase versus one IV administration of bioreactor-produced wildtype recombinant hGLA protein.

We also correlated mouse body weight with peak hGLA serum levels produced at later timepoints in this experiment. In contrast to the close linkage of higher bodyweight to amount of SAHDA-encoded protein production observed in experiment 1, as shown in Fig 1, when hIgG serum levels were measured at day 8 post injection, mouse weight at time of injection did not correlate with peak hGLA serum levels produced by day 49 post-injection an experiment 3 (Fig 3).

As documented previously, the duration of intravenous, single administration HEDGES protein production can be controlled to a significant extent simply by modifying the liposomal lipid composition, the liposomal mean diameter, the DNA vector dose or the specific DNA promoter enhancer element incorporated into the DNA vector [20]. Here, simultaneously co-optimizing different components of the SAHDA platform can substantially control the duration as well as the level of SAHDA-encoded protein production following one SAHDA administration. Specifically, co-optimizing the DNA vector dose, diluent volume, tonicity of the diluent, DNA vector to diluent volume ratio, specific subcutaneous fat sites injected, presence of as well as the dose of hyaluronidase administered, and/or total animal bodyweight can substantially control the level and duration of SAHDA-encoded proteins produced over time.

### Single SAHDA-based administration of a HEDGES hGLA DNA vector produces significantly higher hGLA serum levels over time than single intravenous injection of the identical HEDGES DNA vector

**Fig 4:**
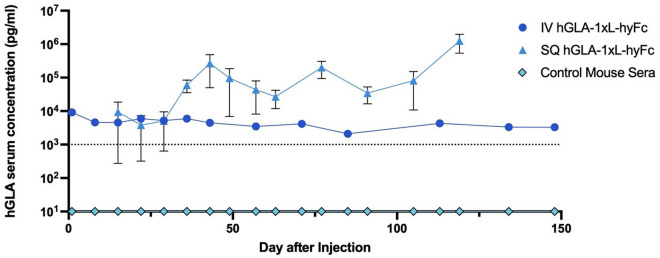
Subcutaneous injection versus intravenous injection. Representative groups of single administration IV-injected mice (n = 4) and single administration SQ-injected mice (n = 5) were assessed over time. Mice were injected and assessed at different times.

Surprisingly, mice receiving one SAHDA-mediated administration of the identical HEDGES wildtype hGLA protein encoding DNA vector commonly produced several orders of magnitude higher serum hGLA levels over time than typical hGLA serum levels produced after one IV HEDGES DNA vector administration (Fig 4). However, while peak hGLA serum levels are produced within 24 hours following one IV HEDGES DNA vector administration, peak hGLA serum levels are produced at significantly later time points (day 22 or later) following one SAHDA-based hGLA protein encoding DNA vector administration. One possible explanation for the differential expression pattern seen with SAHDA versus IV HEDGES could be due related to differences in nuclear DNA entry. It has been hypothesized that the uptake of naked DNA via intravascular routes are through large membrane disruptions, small membrane pores, and endocytosis [28–30]. However, the large membrane disruptions of SAHDA should be much less pronounced than those produced by hydrodynamic injection due to the lack of high volume as well as high-pressure DNA administration. Also, the expression peak does not coincide with the large membrane disruption mechanism which is transient and rapid [28–30].

Additionally, passive entry via small pores requires a longer time for DNA uptake corresponding to the delayed expression of SAHDA. It also cannot be ruled out that unidentified cell surface receptor-mediated entry is at least in part mediated via Stromal Vascular Faction (SVF), a collection of heterogenous cells. SVF are present in approximately two thirds of adipose cells [30]. Committed preadipocytes at various stages appear to be a major portion of SVF that also could contribute to SAHDA mediated subcutaneous adipose cell transfection [30].

### SAHDA based production of protective anti-SARS-CoV2 mAb serum levels

**Fig 5:**
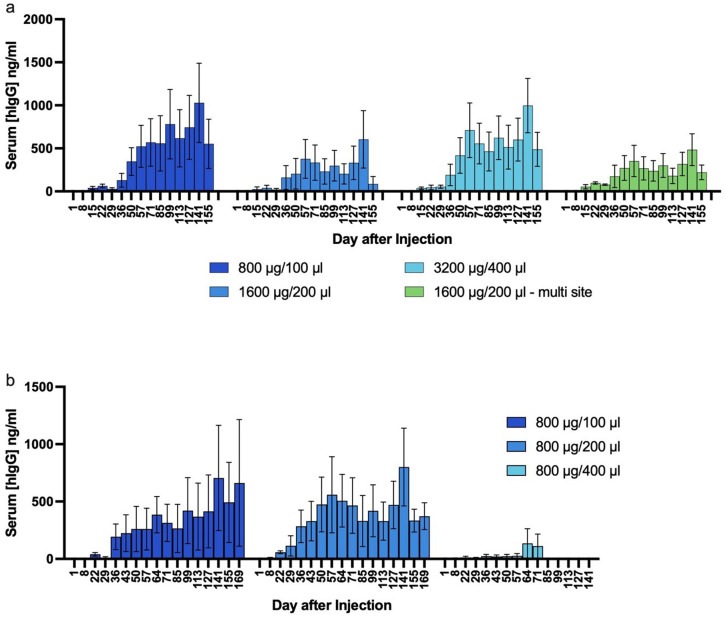
SAHDA driven sustained production of a highly neutralizing anti-SARS-CoV2 mAb serum levels in mice. **a)** Groups of 3 mice were injected with doses of plasmid DNA encoding an anti-SARS-CoV2 mAb (CV07-209) [37] in volumes shown. Mice were injected either once on each side, or in multiple sites per side, as indicated. **b)** Groups of 3 mice were anesthetized and an incision made over the inguinal fat pads. 800 µg of HEDGES DNA vector encoding anti-SARS CoV2 injected in volumes shown. Serum levels of hIgG were assessed over time. Mice in b) were injected and assessed concurrently with mice in **a)**. All data are mean ±SEM.

Previously, we showed that one HEDGES IV injection produces therapeutic serum levels of a broadly neutralizing mAb against 1918 pandemic-influenza (5J8) [22] for over 100 days in mice. Since the outbreak of SARS-CoV2, the rapid mutation of the virus contributed to unsuccessful treatment attempts and continuous pursue of new vaccines or treatments [33–36]. Here we tested whether SAHDA-mediated administration of a DNA vector encoding the anti-SARS-CoV2 mAb (CV07-209) [37] could produce long-term protective serum levels of this anti-SARS-CoV-2 mAb in immunocompetent CD-1 mice. SAHDA-based administration of this anti-SARS-CoV2 mAb (CV07-209) [37] produced prolonged serum levels, reaching > 1 µg/mL (Fig 5a). Additionally, we tested whether dividing the total injection volume into several smaller injection boluses across the mammary fat pad would enhance expression. However, the results were indistinguishable from a single mammary fat pad injection per side (Fig 5b).

### *In vitro* transfection of primary human preadipocytes and adipocytes

**Fig 6:**
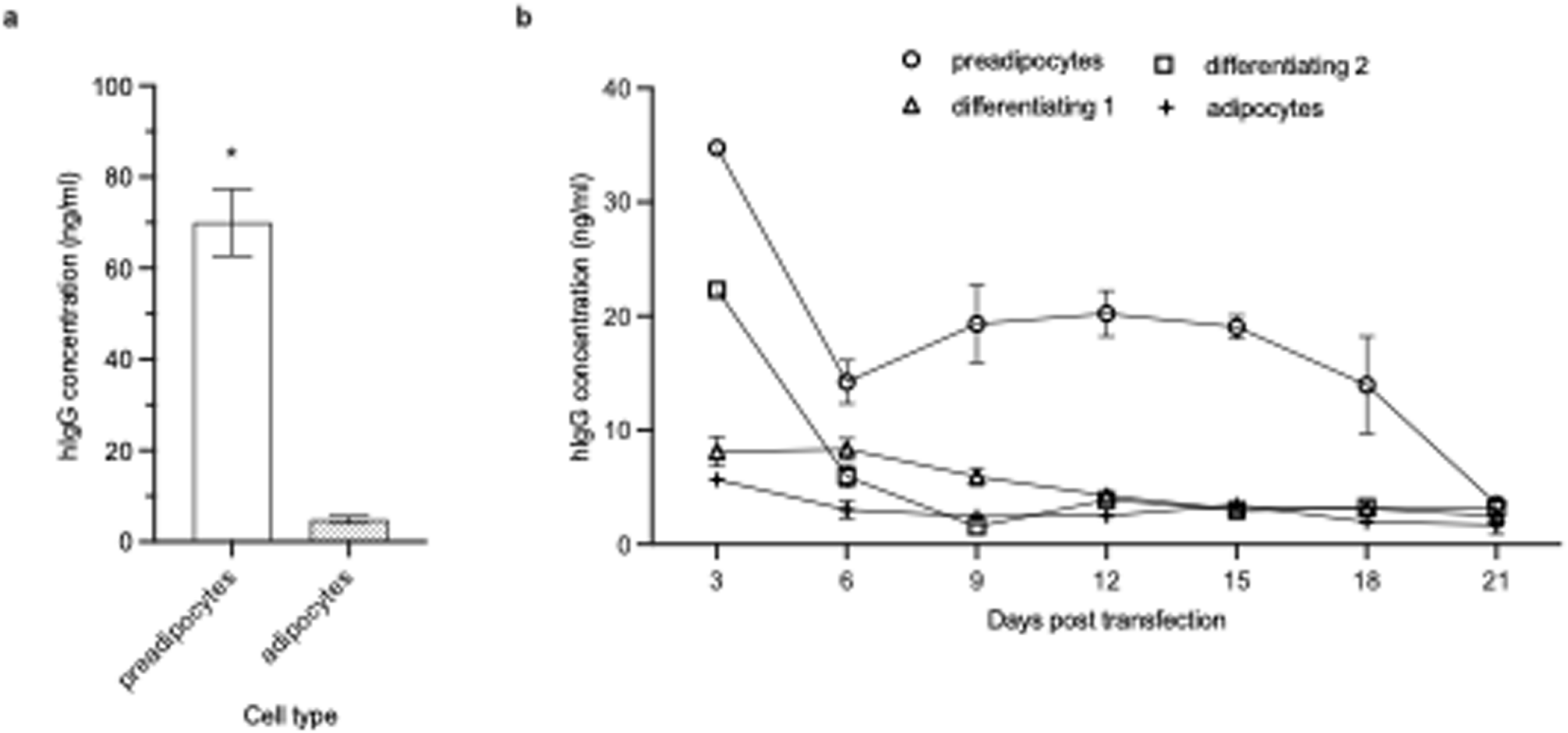
*In vitro* transfection of human primary preadipocytes and adipocytes. **a)** Preadipocyte or adipocyte (shaded) cells were transfected with 1 µg of an anti-SARS-CoV-2 mAb plasmid using 0.54 µL/µg DNA Expifectamine 293. Each sample was collected on day 4 and assayed via hIgG ELISA. Values are mean ±* *SEM (n = 2) and * indicates p-value < 0.05. **b)** Cells were transfected every week throughout the preadipocyte-adipocyte differentiation process, up to day 21. Transfection of differentiating 1 took place on day 6 post initiation of differentiation and differentiating 2 took place on day 15 of differentiation. The supernatant was collected every three days and assayed via hIgG ELISA; values are mean ±* *SEM (n = 2).

Since SAHDA-based HEDGES DNA-vector administration into mice produced durable therapeutic serum levels of cDNA-encoded proteins, we next assessed how efficiently conventional cationic liposome:DNA complexes transfected primary human preadipocytes. We chose to use conventional cationic liposome:DNA complexes because they can efficiently transfect adherent cultured cells overlaid by large volumes of serum containing media. Conversely, plasmid DNA vectors alone cannot transfect cultured cells, which lack cell-surface receptors for DNA alone. To test this, we carried out *in vitro* transfections of primary human white preadipocytes as well as of fully differentiated adipocytes that were differentiated from these preadipocytes. Thin-film transfections were performed with Expifectamine 293 (Thermo Fisher), and the cells were transfected with 1 µg of a HEDGES DNA-vector encoding an anti-SARS-CoV-2 mAb (CV07-209) [37]. Transfection of preadipocytes produced high levels of hIgG levels when compared to the differentiated adipocytes, which displayed low hIgG levels from culture supernatant assayed on day 4 (Fig 6a).

Based on the above results, we tested whether we could identify a specific stage of differentiation from primary preadipocytes to mature adipocytes that was the most highly-transfectable. To accomplish this, separate groups of preadipocytes were plated, then transfected every week following the initiation of differentiation, starting with the primary human preadipocytes and ending with fully mature adipocytes on day 21. Preadipocytes produced significantly higher levels of hIgG compared to each of the other stages of differentiating cells (Fig 6b). Mature adipocytes displayed the lowest hIgG levels in cultures supernatants, as shown by the lower hIgG levels consistently detected from day 3 (5 ng/mL) through day 21. These results are consistent with the results shown in Fig 6a hIgG culture supernatant levels produced by differentiating preadipocytes produced significantly lower hIgG levels when compared to the initial preadipocyte group. However, when compared to the fully differentiated adipocytes, hIgG levels are significantly higher in the differentiating preadipocytes. These results suggest that SAHDA-based preferential targeting of preadipocytes may produce significantly higher levels of SAHDA-encoded proteins following its *in viv*o administration (Fig 6b).

## Discussion

The development of effective and safe, direct subcutaneous infusion of BRPTs has rendered them both safer and less expensive than intravenous infusion [38–40]. However, BRPTs, still exclusively produced *ex vivo* in costly bioreactors, are themselves inherently costly [5]. Therefore, switching to subcutaneous BRPT infusion limits the cost-savings achievable. In addition, some BRPTs must still be administered intravenously, at times posing significant toxicity risks [13,18]. This is a particular problem for individuals suffering from rare monogenic, single protein deficiency diseases, including FD. The high lifetime costs, as well as in some cases the significant toxicity risks of intravenously infusing ERTs remains a persistent worldwide health problem [5,6,14,19]. Therefore, we focused on developing SAHDA as an effective, safe, durable, cost-effective plasmid DNA vector alone-based administration platform. Clearly, a novel platform that can durably functionally replace now absent single monogenic deficiency proteins is urgently required.

Unexpectedly, we discovered that one administration of a SAHDA-based DNA vector encoding the wildtype hGLA protein durably produces at least 100-fold greater hGLA serum levels than high-normal human hGLA 1evels in immunocompetent mice (Fig 4). In addition, one SADHA-based administration of a DNA vector encoding the hGLA protein produces at least 7,200-fold longer hGLA serum levels produced in the normal human range than one wild-type hGLA BRPT administration (serum half-life <20 min) [14]. Importantly, simple modifications to the SAHDA platform can control the level as well as the duration of hGLA protein production produced over a broad temporal range in immunocompetent mice. Specifically, co-optimizing the DNA vector dose, diluent volume, tonicity of the diluent, DNA vector to diluent volume ratio, specific subcutaneous fat sites injected, presence of as well as the dose of hyaluronidase administered, and/or total animal bodyweight can significantly control the level and duration of SAHDA-encoded proteins produced over time. Furthermore, SAHDA-based administration of a DNA vector encoding the highly neutralizing anti-SARS-CoV-2 mAb (CV07-209) [37] produces hIgG serum levels approaching 1 mg/mL for greater than 100 mouse days.

A major BRPT disadvantage is that all recombinant protein therapies, including ERT, are produced *ex vivo* in costly bioreactors [41]. Conversely, one SAHDA administration obviates the need for bioreactor generated recombinant protein production. Rather, one SAHDA administration, following an initial delay, then continuously produces the cDNA encoded transgene protein(s) within each person’s own fat cells. These transfected fat cells then durably secrete therapeutic levels of these cDNA encoded therapeutic transgene proteins directly into the systemic circulation. Approximately 17–30% of preadipocytes are present in human subcutaneous fat [42]. This is of critical importance, as Fig 6 documents that primary human preadipocytes are much more efficiently transfected than differentiating primary human adipocytes. This could enable durable, cost-effective production of SAHDA-encoded proteins in very large numbers of SAHDA-treated individuals worldwide. Furthermore, unlike BRPTs, SAHDA does not require refrigeration, can readily be freeze-dried and is readily self-administered [43].

In addition, SAHDA’s safety as well as cost-efficacy profiles offer significant advantages over other protein producing platforms. Specifically, hydrodynamic administration of plasmid DNA alone in animals has produced minimal toxicity while producing persistent serum levels of biologically active proteins [44]. Furthermore, IV injecting 2 mg/kg of cationic liposome:DNA complexes produced detectable mouse toxicity, whereas injecting 33 mg/kg of the identical plasmid DNA alone produced no detectable mouse toxicity overtime [45]. Last, mRNA nanoparticles encoding an mRNA-based vaccine were injected with an MTD of 0.6 mg/kg as a single intravenous infusion into healthy human beings [46]. Subsequently, 21.4% of patients experienced a grade 2 infusion reaction, 2.7% experienced a grade 3 infusion reaction. Conversely, in mice receiving a 160 mg/kg dose of a SAHDA-based DNA vector alone encoding the hGLA protein. no detectable adverse effects observed in any of these mouse for more than the next 175 days (Fig 4). Therefore, the mRNA:nucleoprotein complex had an MTD of 0.6 mg/kg [46]. Conversely, a 160 mg per kg SAHDA administration produced no detectable toxicity. Thus, mRNA:nucleoprotein complexes were greater than 333 fold more toxic than SAHDA. Clearly, these two groups are not strictly complementary. However, it does appear clear that plasmid DNA alone does not efficiently elicit innate immune responses compared to when DNA or RNA is presented as a PAMP in the context of a microbial cell membrane or wall [47].

Last, for all SAHDA studies performed, we exclusively used immunocompetent mice. As shown previously, one IV administration of a HEDGES DNA vector encoding rituximab [23], a largely humanized, FDA-approved mAb, elicits highly neutralizing mouse anti-human protein antibody responses in up to one third of IV injected immunocompetent mice over time [20]. The production of these highly neutralizing mouse anti-human antibody responses produces an interspecies artifact that then rapidly reduces rituximab serum protein levels to background levels [20]. Therefore, SAHDA-based administration of fully human proteins should prove more effective in humans than mice (Table 1).

**Table 1:** Direct comparison of SAHDA vs BRPT vs AAV.

Critical parameter	SAHDA	BRPTs	AAV
Duration of hGLA serum protein levels in the normal 1–10 ng/mL human range produced following one administration	Durable^?^	Every 2 weeks [2]	Durable [15]
Can be cffectively re-dosed	Yes [20]	Yes [2]	No [17]
Causes life-threatening allergic infusion reactions	No [24]	Yes [9]	No [17]
Can elicit fatal adaptive immune responses	No [24]	No [9]	Yes [17]
Produces durable therapeutic levels of different protein therapies	Yes [24]	No [9]	Yes [15]
Produced *in vivo or ex vivo*	*In vivo* [24]	*Ex vivo* [9]	*In vivo* [15]
Detectably integrates into genomic DNA	No [24]	No [9]	Yes [20]
Requires an intact cold chain	No [24]	Yes [9]	Yes [15]
Can be freeze dried	Yes [24]	No [9]	Yes [29]

Taken together, our results suggest that one SAHDA administration may effectively, durably, safely, and cost-effectively treat a spectrum of now difficult or impossible to treat human diseases, even in the world’s most under resourced areas. Thus, it is possible SAHDA may progressively more effectively, safely, durably, and cost-effectively treat rare, human monogenic single protein deficiency diseases worldwide.
